# Functional and Behavioral Restoration of Vision by Gene Therapy in the Guanylate Cyclase-1 (GC1) Knockout Mouse

**DOI:** 10.1371/journal.pone.0011306

**Published:** 2010-06-25

**Authors:** Shannon E. Boye, Sanford L. Boye, Jijing Pang, Renee Ryals, Drew Everhart, Yumiko Umino, Andy W. Neeley, Joseph Besharse, Robert Barlow, William W. Hauswirth

**Affiliations:** 1 Department of Ophthalmology, College of Medicine, University of Florida, Gainesville, Florida, United States of America; 2 Department of Ophthalmology, College of Medicine, State University of New York (SUNY) Upstate Medical University, Syracuse, New York, United States of America; 3 Department of Cell Biology, Neurobiology and Anatomy, Medical College of Wisconsin, Milwaukee, Wisconsin, United States of America; Universidade Federal do Rio de Janeiro (UFRJ), Brazil

## Abstract

**Background:**

Recessive mutations in guanylate cyclase-1 (*Gucy2d*) are associated with severe, early onset Leber congenital amaurosis-1(LCA1). *Gucy2d* encodes guanylate cyclase (GC1) is expressed in photoreceptor outer segment membranes and produces cGMP in these cells. LCA1 patients present in infancy with severely impaired vision and extinguished electroretinogram (ERG) but retain some photoreceptors in both their macular and peripheral retina for years. Like LCA1 patients, loss of cone function in the GC1 knockout (GC1KO) mouse precedes cone degeneration. The purpose of this study was to test whether delivery of functional GC1 to cone cells of the postnatal GC1KO mouse could restore function to these cells.

**Methodology/Principal Findings:**

Serotype 5 AAV vectors containing either a photoreceptor-specific, rhodopsin kinase (hGRK1) or ubiquitous (smCBA) promoter driving expression of wild type murine GC1 were subretinally delivered to one eye of P14 GC1KO mice. Visual function (ERG) was analyzed in treated and untreated eyes until 3 months post injection. AAV-treated, isogenic wild type and uninjected control mice were evaluated for restoration of visual behavior using optomotor testing. At 3 months post injection, all animals were sacrificed, and their treated and untreated retinas assayed for expression of GC1 and localization of cone arrestin. Cone-mediated function was restored to treated eyes of GC1KO mice (ERG amplitudes were ∼45% of normal). Treatment effect was stable for at least 3 months. Robust improvements in cone-mediated visual behavior were also observed, with responses of treated mice being similar or identical to that of wild type mice. AAV-vectored GC1 expression was found in photoreceptors and cone cells were preserved in treated retinas.

**Conclusions/Significance:**

This is the first demonstration of gene-based restoration of both visual function/vision-elicited behavior and cone preservation in a mammalian model of GC1 deficiency. Importantly, results were obtained using a well characterized, clinically relevant AAV vector. These results lay the ground work for the development of an AAV-based gene therapy vector for the treatment of LCA1.

## Introduction

Leber congenital amaurosis (LCA) is an autosomal recessive group of diseases that represent the earliest and most severe form of all inherited retinal dystrophies. The first gene implicated in the onset of this genetically and clinically heterogeneous disease, and therefore assigned to the LCA1 locus was retinal-specific *Guanylate cyclase-1* (*Gucy2d*) [1]. *Gucy2d* encodes the retina- specific protein guanylate cyclase (GC1) which is expressed in both cone and rod photoreceptor disc membranes [2]–[3] and plays a role in the regulation of cGMP and Ca^2+^ levels within these cells. Following light stimulation, levels of cGMP within photoreceptor outer segments rapidly fall due to hydrolysis by cGMP phosphodiesterase (PDE). This reduction of cGMP leads to a closure of cGMP-gated channels, reduced Ca^2+^ influx and hyperpolarization of the cell. This decrease in intracellular Ca^2+^ stimulates return of light-stimulated photoreceptors to the dark state via its interaction with guanylate cyclase (GC) activating proteins (GCAPs), a family of calcium binding proteins that regulate the activity of GC. In the dark adapted photoreceptor, Ca^2+^ bound GCAPs inhibit the activity of GC. Upon light stimulation, however, Ca^2+^-free GCAPs stimulate GC activity which increases cGMP levels, reopens cGMP-gated channels and a returns the cell to a depolarized state [4]. Mutations which reduce or abolish the ability of GC to replenish intracellular cGMP and reopen cGMP-gated cation channels, as is the case in LCA1, are thought to create the biochemical equivalent of chronic light exposure in rod and cone photoreceptors [5].

Mutations in *Gucy2d* account for as many as 20% of all cases of LCA making it one of the leading causes of this disease [1],[6],[7] The number of patients affected by LCA1 is approximately double that affected by the well known RPE65 version of LCA (LCA2) [8],[9]. Diagnosis of LCA1 is typically made within the first few months of life and is characterized by severely impaired vision, extinguished electroretinogram (ERG) and pendular nystagmus [8], [10]. Despite these functional deficits, LCA1 patients present with normal fundus [8] and retain some rods and cones in both their macular and peripheral retina for years [6], [11]–[12]. Using spectral-domain optical coherence tomography (SDOCT) to scan the central macular and perifoveal areas, a recent study revealed that LCA1 patients (age range, 20–53 years) retained all 6 retinal layers with a visible photoreceptor inner/outer segment juncture [12]. Maintenance of retinal structure in LCA1 is unlike other forms of the disease which exhibit marked retinal thinning that generally worsens with age [12]. While the preservation of retinal structure does not parallel better visual acuity in LCA1 patients, it does suggest that they may be responsive to gene-based therapeutic strategies that require some level of rod/cone cell preservation.

Two animal models carrying null mutations in the GC1 gene have been used to evaluate gene replacement therapy, the naturally occurring GUCY1*B chicken and the guanylate-cyclase-1 (GC1) knockout mouse [13]–[14]. The GUCY1*B chicken is blind at hatch, exhibits extinguished scotopic (rod-mediated) and photopic (cone-mediated) ERG and retinal degeneration [5], [15]–[16]. Prehatch lentiviral-mediated transfer of *Gucy2d* to the GUCY1*B retina restored vision to these animals as evidenced by behavioral testing and ERG analysis [13]. Despite the short term therapeutic success, this therapy fell short of preserving retinal structure or function in the long term. The transient nature of this result, obtained in a non-mammalian species with an integrating viral vector delivered *in-ovo* suggested the need for more appropriate translational studies towards the development of clinical application. A mammalian model of GC1 deficiency, the GC1KO mouse exhibits cone photoreceptor degeneration [17]–[18]. Like LCA1 patients, loss of cone function in this mouse model precedes cone degeneration [17]. Rod photoreceptors in this model do not degenerate and continue to generate ERG responses to light [17], a result likely due to the presence of GC2, a close relative of GC1 in these cells [19]–[22]. AAV-mediated transfer of *Gucy2d* to the post-natal GC1KO retina failed to restore cone ERG responses or prevent cone degeneration [14]. In both the chicken and mouse studies which were conducted by the same investigators, the therapeutic cDNA was of bovine origin, the species historically used in biochemical assays evaluating GC1 function [13], [23]. This raises the question of whether the heterologous nature of these gene transfer strategies was the reason for their incomplete success.

In the present series of experiments, we evaluated whether delivery of a species-specific (murine) version of GC1 to cones of the postnatal GC1KO mouse could restore function to and preserve these cells. Serotype 5 AAV vectors were used to deliver mGC1 to photoreceptors of postnatal day 14 (P14) GC1KO mice. The results reported herein are the first demonstration that gene therapy is capable of restoring visual function and visually evoked behavior to a mammalian model of GC1 deficiency. Importantly, results were obtained following postnatal delivery of a well characterized, clinically relevant AAV vector. These results lay the groundwork for the development of an AAV-based gene therapy vector for treatment of LCA1.

## Materials and Methods

### Experimental Animals

GC1+/− heterozygote embryos were removed from a cryopreserved stock at The Jackson Laboratory (Bar Harbor, ME). Heterozygotes were mated at the University of Florida to produce GC1 KO (−/−) and isogenic +/+ control offspring. All mice were bred and maintained in the University of Florida Health Science Center Animal Care Services Facility under a 12hr/12hr light/dark cycle. Food and water were available ad libitum. All experiments were approved by the University of Florida's Institutional Animal Care and Use Committee and conducted in accordance with the ARVO Statement for the Use of Animals in Ophthalmic and Vision Research and NIH regulations.

### Construction of AAV vectors

Serotype 5 Adeno-associated virus (AAV5) vectors were used to deliver murine GC1 (mGC1) as they have been shown to exhibit robust transduction efficiency and relatively quick expression in retinal photoreceptors [24]. Both a cell-specific and ubiquitous promoter were selected to drive expression of mGC1 (generously provided by Joseph Besharse). A promoter for the cell-specific, G protein-coupled receptor kinase 1 (GRK1), also known as rhodopsin kinase was chosen for its ability to specifically target robust transgene expression in rod and cone photoreceptors when used in conjunction with AAV [25]. The ubiquitous smCBA promoter which exhibits a similar expression pattern to full-length CBA in retina was chosen for its ability to efficiently target the neural retina [14]. Polymerase chain reaction utilizing forward primer 5′AAAAGCGGCCGCATGAGCGCTTGGCTCCTGCCAGCC3′ and reverse primer 5′ AAAAGCGGCCGCTCACTTCCCAGTAAACTGGCCTGG3′ was used to amplify mGC1 from a plasmid containing an mGC1-eGFP fusion [26]. The resulting fragment was cloned into pCRblunt plasmid (Invitrogen, Carlsbad, CA) and then sequence verified. AAV vector plasmid containing smCBA driving expression of mGC1 (pTR-smCBA-mGC1) was created by replacing full length CBA with smCBA in plasmid pTR-CB^SB^-hRPE65 [27] via EcoR I digest and ligation. Subsequently, hRPE65 was replaced with mGC1 via Not I digestion and ligation, resulting in the creation of pTR-smCBA-mGC1. AAV vector plasmid containing human GRK1 promoter driving expression of mGC1, pTR-GRK1-mGC1 was created by removing hGFP from pTR-hGRK1-hGFP [28] and replacing it with mGC1 via Not I digest and ligation. AAV vectors were packaged according to previously published methods [14]. Viral particles were resuspended in Balanced Salt Solution (Alcon, Fort Worth, TX) and titered by quantitative real-time PCR [27]. Resulting titers were 4.69×10^12^ vg/ml and 4.12×10^13^ vg/ml for AAV5-smCBA-mGC1 and AAV5-hGRK1-mGC1, respectively.

### Subretinal Injections

One µl of AAV5-GRK1-mGC1 (4.12×10^10^ delivered vector genomes) or AAV5-smCBA-mGC1 (4.69×10^9^ delivered vector genomes) was injected subretinally at postnatal day 14 (P14) to the right eye of each GC1KO mouse, leaving the left eye as a contralateral control. Subretinal injections were performed as previously described [29]–[30]. Further analysis was carried out only on animals which received comparable, successful injections (>60% retinal detachment and minimal complications). It is well established that the area of retinal detachment corresponds to the area of viral transduction [29], [31].

### Electroretinographic analysis

Electroretinograms of treated GC1KO (n = 14) and isogenic +/+ controls (n = 2) were recorded using a PC-based control and recording unit (Toennies Multiliner Vision; Jaeger/Toennies, Höchberg, Germany) according to methods previously described with minor modifications [14]. Initial ERG measurements were recorded at 4 weeks post injection, and every subsequent 2 weeks thereafter until 3 months post-injection (the latest time point evaluated in this study). Age matched +/+ isogenic controls were recorded alongside treated animals at every time point. Mice were dark-adapted overnight (more than 12 hours) and anesthetized with a mixture of 100 mg/kg ketamine, 20 mg/kg xylazine and saline in a 1∶1∶5 ratio, respectively. Pupils were dilated with 1% tropicamide and 2.5% phenylephrine hydrochloride. A heated circulating water bath was used to maintain the body temperature at 38°C. Hydroxypropyl methylcellulose 2.5% was applied to each eye to prevent corneal dehydration. Full field ERGs were recorded using custom, gold wire loop corneal electrodes. Reference and ground electrodes were placed subcutaneously between the eyes and in the tail, respectively. Scotopic rod recordings were elicited with a series of white flashes of seven increasing intensities (.01 mcds/m^2^ to 5 cds/m^2^). Interstimulus intervals for low intensity stimuli were 1.1 second. At the three highest intensities (100 mcds/m^2^, 1 cds/m^2^ and 5 cds/m^2^), instimulus intervals were 2.5, 5.0 and 20.0 seconds, respectively. Ten responses were recorded and averaged at each intensity. Mice were then light adapted to a 100 cds/m^2^ white background for 2 minutes. Photopic cone responses were elicited with a series of five increasing light intensities (100mcds/m^2^ to 12 cds/m^2^). Fifty responses were recorded and averaged at each intensity. All stimuli were presented in the presence of the 100 cds/m^2^ background. B-wave amplitudes were defined as the difference between the a-wave troughs to the positive peaks of each waveform. Photopic b-wave maximum amplitudes (those generated at 12 cds/m^2^) of all smCBA-mGC1- treated (n = 6) and hGRK1-mGC1- treated (n = 8) GC1KO (both treated and untreated eyes) and isogenic +/+ control mice were averaged and used to generate standard errors. These calculations were made at every time point (4 weeks-13 weeks post injection). This data was imported into Sigma Plot for final graphical presentation. The paired *t*-test was used to calculate *P*-values between treated and untreated eyes within each promoter group (smCBA or hGRK1) and between each promoter group over time (4 weeks post-injection vs. 3 months post-injection). The standard *t*-test was used to calculate *P*-values between smCBA-mGC1 vs. hGRK1-mGC1 treated eyes. Significant difference was defined as a *P*-value<0.05. Because a subset of mice from each treated group was sent to SUNY Upstate for behavioral analyses, the total number of mice averaged and presented at each time point in results differs. Three mice from the smCBA-mGC1-treated group were sent for optomotor testing, leaving an *n* of 3 mice used for ERG analysis during the 8, 10 and 12 week measurements. Two mice from the hGRK1-mGC1- treated group were sent for optomotor testing, leaving an *n* of 6 used for ERG analysis during the 6, 8, 10 and 12 week measurements. All mice sent for behavioral analysis were analyzed subsequently by ERG at 13 weeks post injection upon their return to the University of Florida (smCBA-mGC1: n = 3, hGRK1-mGC1: n = 2).

### Optomotor Testing

Photopic visual acuities and contrast sensitivities of treated and untreated GC1KO mouse eyes were measured using a two-alternative forced choice paradigm as described previously [32],[33]. To test the sensitivity of individual eyes from the same animal we took advantage of the fact that mouse vision has minimal binocular overlap and that the left eye is more sensitive to clockwise rotation and the right to counter-clockwise rotation [34]. Thus in our “randomize-separate” optomotor protocol, each eye's acuity and contrast sensitivity threshold was determined separately and simultaneously via stepwise functions for correct responses in both the clockwise and counter-clockwise directions. Correct detection of patterns rotating in the clockwise direction was driven primarily by visual signals originating from the left eye and correct responses in the counterclockwise direction were derived from visual signals originating from the right eye. Acuity was defined the highest spatial frequency (100% contrast) yielding a threshold response, and contrast sensitivity was defined as 100 divided by the lowest percent contrast yielding a threshold response. For photopic acuity, the initial stimulus was a 0.200 cycles/degree sinusoidal pattern with a fixed 100% contrast. For photopic contrast sensitivity measurements, the initial pattern was presented at 100% contrast, with a fixed spatial frequency of 0.128 cycles/degree. Photopic vision was measured at a mean luminance of 70 cd/m^2^. Visual acuities and contrast sensitivities were measured for both eyes of each mouse four to six times over a period of 1 week. Age matched, isogenic +/+ control animals (M1, M2) and naïve GC1KO mice (M3, M4) are presented along with the smCBA-mGC1-treated (M5, M6, M7) and hGRK1-mGC1-treated mice (M8, M9). Cone-mediated ERG amplitudes generated from a 12 cds/m^2^ stimulus of all mice (M1–M9) are presented alongside the behavior results. Unpaired t-tests were carried out on acuity and percent contrast values to determine significance of results.

### Tissue Preparation

Three months post injection, P14-treated GC1KO mice were sacrificed. The limbus of injected and uninjected eyes was marked with a hot needle at the 12 o'clock position, facilitating orientation. Enucleation was performed under dim red light and eyes were placed immediately in 4% paraformaldehyde. Eyes that were to be used for cryosectioning were prepared according to previously described methods [14]. Briefly, corneas were removed from each eye, leaving the lens inside the remaining eye cup. A small “V” shaped cut was made into the sclera adjacent to the burned limbus to maintain orientation. After overnight fixation, the lens and vitreous were removed. The remaining retina/RPE-containing eyecup was placed in 30% sucrose in PBS for at least 1 hour at 4°. Eyecups were then placed in cryostat compound (Tissue Tek OCT 4583; Sakura Finetek USA, Inc., Torrance, CA) and snap frozen in a bath of dry ice/ethanol. Eyes were serially sectioned at 10 microns with a cryostat (Microtome HM550; Walldorf, Germany). Eyes that were to be used for whole mount analysis were prepared according to previously described methods [35]. Orientation was achieved as previously mentioned. After overnight fixation, cornea, lens, vitreous and retinal pigment epithelia were removed from each eye without disturbing the retina. A cut was made in the superior (dorsal) portion of the retina adjacent to the original limbus burn to maintain orientation.

### Immunohistochemistry and Microscopy

Retinal cryosections and whole mounts were washed 3 times in 1× PBS. Samples were then incubated in 0.5% Triton X-100 for 1 hour in the dark at room temperature, blocked in a solution of 1% bovine serum albumin (BSA) in PBS for 1 hour at room temperature and incubated overnight at 37° with a rabbit polyclonal GC1 antibody (1∶200, sc-50512, Santa Cruz Biotechnology, Inc.), rabbit polyclonal cone arrestin antibody (“Lumij” 1∶1000, generously provided by Dr. Cheryl Craft) or a lectin PNA conjugated to Alexa Fluor 488 (1∶200, L21409, Invitrogen) diluted in 0.3% Triton X-100/1% BSA. Retinal whole mounts were incubated overnight at room temperature with the same cone arrestin antibody, diluted 1∶1000 in 0.3% Triton X-100/1% BSA. Following primary incubation, retinal sections and whole mounts were washed 3 times with 1× PBS then incubated for 1 hour at room temperature with IgG secondary antibodies tagged with either Alexa-594 or Alexa-488 fluorophore (Molecular Probes, Eugene OR) diluted 1∶500 in 1× PBS and washed with 1× PBS. Retinal sections were counterstained with 4′, 6′-diamino-2-phenylindole (DAPI) for 5 minutes at room temperature. After a final rinse with 1× PBS and water, sections were mouted in an aqueous-based media (DAKO) and coverslipped. Retinal whole mounts were oriented on slides with the superior (dorsal) portion of the retina positioned at 12- o'clock. Samples were mounted in DAKO and coverslipped. Retinal sections were analyzed by confocal microscopy (Leica TCS SP2 AOBS Spectral Confocal Microscope equipped with LCS Version 2.61, Build 1537 software). All images were taken with identical exposure settings at either 20× or 63× magnification. Excitation wavelengths used for DAPI, GC1 and cone arrestin stains were 405, 488 and 594, respectively. Emission spectra were 440–470nm, 500–535nm and 605–660nm, respectively. Retinal whole mounts were analyzed with a widefield fluorescent microscope (Zeiss Axioplan 2) equipped with a QImaging Retiga 4000R Camera and QImaging QCapture Pro software. Quadrants of each whole mount were imaged at 5× under identical exposure settings and then merged together in Adobe Photoshop.

### Image Analysis

Cone photoreceptor densities were analyzed in retinal whole mounts by counting cells labeled with secondary fluorophore directed against cone arrestin antibody in the central and inferior retina using ImageJ software (NIH, Bethesda, MD, USA). Five squares (500 µm^2^) were placed over identical areas in central and inferior retina of both treated and untreated GC1KO eyes. For central retina, squares were placed at an equal eccentricity around the optic nerve head in all eyes (125 µm). Cone photoreceptors were counted in each respective retinal area, values were averaged and standard deviations calculated. The standard t-test was used to calculate *P*-values between desired samples. Significant difference was defined as a *P*-value<0.05.

## Results

### Both the photoreceptor-specific human RK promoter and ubiquitous smCBA promoter drive mGC1 transgene expression in rods and cones of GC1KO mice

**Figure 1:**
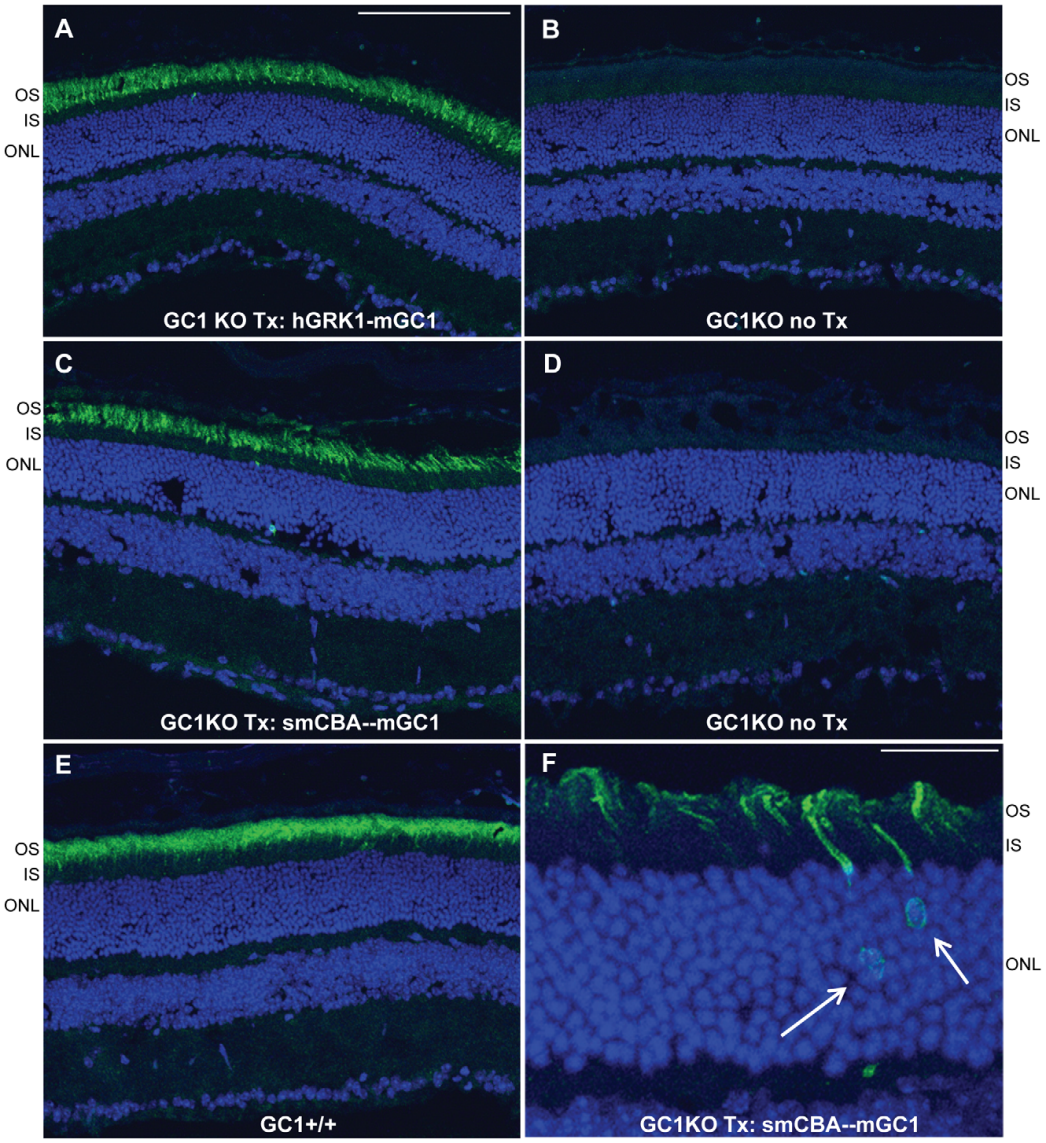
AAV-mediated GC1 expression in photoreceptors of the GC1KO mouse. AAV5-hGRK1-mGC1 drives expression of GC1 in photoreceptor outer segments of GC1KO mice. (A). No GC1 expression is seen in the untreated, contralateral control eye (B). AAV5-smCBA-mGC1 drives expression of GC1 in photoreceptor outer segments (C) and occasionally in photoreceptor cell bodies (arrows in F). No such GC1 expression is seen in the untreated, contralateral control eye (D). Levels of therapeutic transgene expression in the AAV5-mGC1-treated eyes are only slightly less than that seen in isogenic GC1+/+ control eyes (E). All retinas were taken from mice 3 months post treatment or age matched untreated controls. Scale bars in A = 100µm, F = 25µm. OS-outer segments, IS-inner segments, ONL-outer nuclear layer.

**Figure 2:**
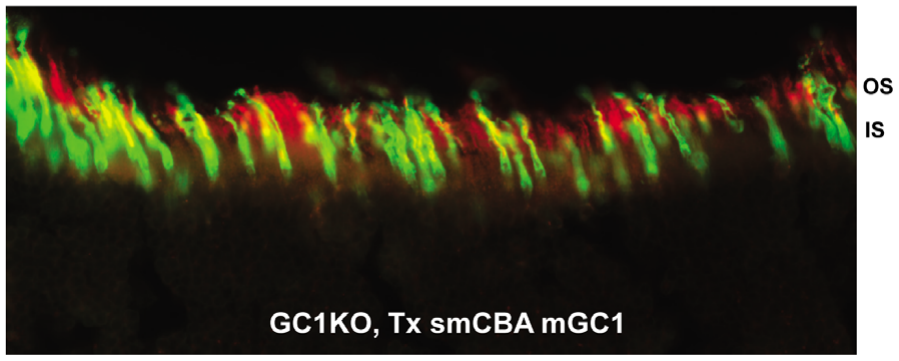
AAV5-mGC1 drives expression of GC1 in both rod and cone photoreceptors. Representative retinal section from a GC1KO eye injected with AAV5-smCBA-mGC1 stained for GC1 (red) and PNA lectin (green) reveals GC1 expression in cone outer segments (yellow overlay) as well as in rod outer segments (red alone). hGRK1-mGC1 injected eyes revealed the same pattern (data not shown).

GC1- deficiency affects both rod and cone photoreceptors in LCA1 patients. We therefore chose the photoreceptor-specific human RK promoter and the ubiquitous smCBA promoter for this study as a means to target both cell types. The human RK promoter was chosen for its small size and ability to efficiently drive transgene expression specifically in photoreceptor cells [25], [36]–[37]. Immunostaining of GC1KO retinas 3 months post-treatment with AAV5-hGRK1-mGC1 revealed that this promoter drove robust GC1 expression exclusively in photoreceptor (rod and cone) outer segments. A representative image of a retinal cross section from an eye injected with this therapeutic vector (Figure 1A) shows intense GC1 staining in the OS layer whereas the contralateral, untreated eye lacks any GC1 expression (Figure 1B). The smCBA promoter also efficiently drove GC1 expression in photoreceptor cells. Photoreceptor OS exhibited robust smCBA-mediated GC1 expression in treated eyes (Figure 1C), relative to the contralateral, untreated eye (Figure 1D). Levels of hGRK1 and smCBA-mediated GC1 expression approached those seen in isogenic, +/+ control eyes (Figure 1E). GC1 expression in hGRK1-mGC1-treated eyes was restricted to outer segments. In smCBA-mGC1- treated eyes, GC1 expression was occasionally found in photoreceptor inner segments and cell bodies of the outer nuclear layer (Figure 1F, arrows). Notably however, neither promoter construct drove therapeutic GC1 expression in retinal cells other than photoreceptors. This lack of off-target expression could be relevant to the development of safe future clinical applications. A representative section stained for GC1 was also stained with lectin PNA which labels cone outer segment sheaths. Figure 2 shows an overlay of GC1 (red) and PNA (green) expression in cone cells. GC1 expression was not exclusive to cones, however, proving that therapeutic transgene is expressed in both rods and cones of treated GC1KO mice (Figure 2).

### Photoreceptor function (ERG) is restored in AAV-treated GC1KO mice

**Figure 3:**
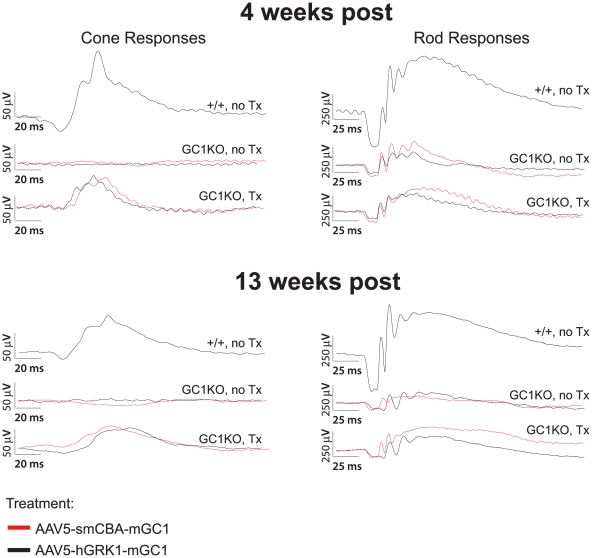
AAV5-mGC1 restores retinal function to cone photoreceptors of the GC1KO mouse. Representative cone (left column)- and rod (right column)-mediated ERG traces from GC1 +/+ (upper waveforms), untreated GC1KO (middle waveforms) and AAV5-mGC1-treated (bottom waveforms) mice. For the middle and bottom waveforms in each panel, red traces correspond to eyes injected with AAV5-smCBA-mGC1 (bottom) and their uninjected contralateral eyes (middle) and black traces correspond to eyes injected with AAV5-hGRK1-mGC1 (bottom) and uninjected contralateral eyes (middle). Cone responses in AAV5-mGC1-treated eyes are restored to approximately 45% of normal amplitude.

**Figure 4:**
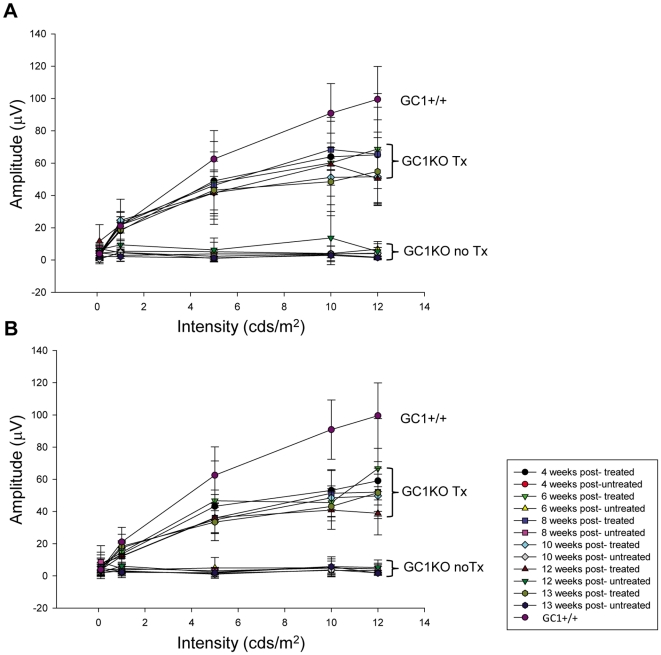
Average photopic b-wave maximum amplitudes as a function of both flash intensity and time after treatment. Responses of GC1KO, isogenic +/+ controls, AAV5-smCBA-mGC1-treated (A) and AAV5-hGRK1-mGC1-treated (B) GC1KO mice reveal that cone responses in both smCBA-mGC1 and hGRK1-mGC1-treated mice are approximately 45% of normal for at least 3 months post injection (the longest time point evaluated in this study).

It was previously reported that cone responses in the GC1KO mouse are barely detectable by 1 month of age [17]. Here we show that P14-treatment of this mouse with an AAV vector carrying the mouse GC1 gene under the control of either a photoreceptor-specific (hGRK1) or ubiquitous (smCBA) promoter led to substantial restoration of cone photoreceptor function as measured by ERG. Representative cone traces (Figure 3A) as well as the average photopic b-wave amplitudes (Figure 4) from hGRK1-mGC1-treated, smCBA-mGC1-treated, GC1KO and isogenic +/+ controls show that cone function in treated eyes is restored to approximately 45% of normal at four weeks post injection. Similar to previous reports, cone responses in contralateral, untreated eyes were undetectable at this time point. At 4 weeks post injection, the average cone-mediated b-wave amplitude in smCBA-mGC1-treated eyes (65.1 µV) was significantly higher (*P* = .006) than that in the untreated eyes (3.9 µV). The average cone mediated b-wave amplitude in hGRK1-mGC1-treated eyes (59.1 µV) was significantly higher (*P*<.001) than that in untreated eyes (3.2 µV). The level of restoration achieved four weeks following delivery of the photoreceptor-specific hGRK1-mGC1 vector was not significantly different from that achieved with the ubiquitous promoter-containing smCBA-mGC1 vector (*P* = .604). At 3 months post injection, the average cone-mediated b-wave amplitude in smCBA-mGC1-treated eyes (53.3 µV) was significantly higher (*P*<.001) than that in the untreated eyes (2.8 µV). The average cone mediated b-wave amplitude in hGRK1-mGC1-treated eyes (45.3 µV) was significantly higher (*P*<.001) than that in untreated eyes (3.4 µV). The level of restoration achieved 3 months following delivery of the photoreceptor-specific GRK1-mGC1 vector was not significantly different from that achieved with the ubiquitous promoter-containing smCBA-mGC1 vector (*P* = .331). Both promoters conferred similar levels of functional restoration to cones in treated eyes of the GC1KO mouse in the short term. Importantly, restoration of cone photoreceptor function remained stable for 3 months (the latest time point evaluated in this study) (Figures 3 and 4). There was no significant difference in photopic b-wave amplitudes of smCBA-mGC1-treated or hGRK1-mGC1-treated eyes between 4 weeks and 3 months post treatment (*P* = 0.174 and 0.125, respectively).

ERG implicit times which are an important feature in the diagnosis of various retinal disorders including other forms of LCA [36] were also determined. While such measurements cannot be reliably obtained from an untreated GC1KO eye, we were able to compare cone b-wave implicit times in AAV-mGC1 treated and isogenic +/+ control mice. At 4 weeks post injection, there was no significant difference between cone b-wave implicit times in treated and +/+ control eyes (*P* = 0.884); average values in AAV-mGC1-treated and +/+ eyes at this time point were 50.8 ms and 50.4 ms, respectively. At 3 months post injection, there was also no significant difference between the two groups (*P* = 0.697); averages of all cone b-wave implicit times in treated and +/+ control eyes were 59.7 ms and 58.3 ms, respectively. The response kinetics of cones in the treated GC1KO retina, as determined by implicit time measurements appeared to be normal and stable in the short term.

It was previously reported that rod ERGs in the GC1KO mouse show alterations by 1 month of age, with the rod a-wave and b-wave both markedly reduced [17]. This loss plateaus at 5 months of age with responses approximately 50–70% that of a WT mouse. While we observed some instances of AAV-mGC1-mediated improvements in treated eyes of GC1KO mice relative to untreated controls (example seen in Figure 3), this result was not as consistent as that seen for the cone-mediated responses. Additional studies are required to elucidate how effective this therapy will be at restoring rod-mediated ERGs.

### Cone mediated visual acuity and contrast sensitivity are restored in AAV-treated GC1KO mice

**Figure 5:**
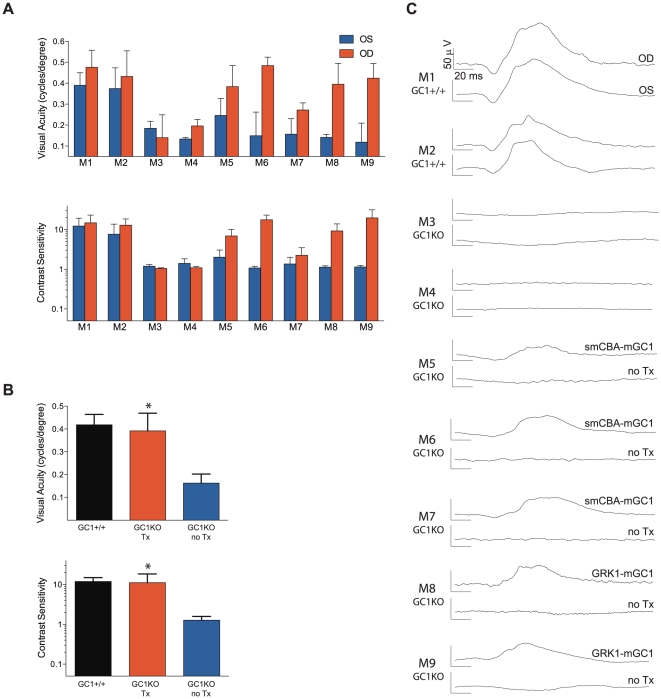
Optomotor analysis of visual function restoration in GC1KO mice treated with either AAV5-smCBA-mGC1 or AAV5-hGRK1-mGC1. M1–M9 correspond to the nine mice used for testing. Photopic acuities and contrast sensitivities of GC1+/+ control mice (M1, M2), naïve GC1KO (M3, M4), smCBA-mGC1-treated (M5, M6, M7) and hGRK1-mGC1-treated GC1KO (M8, M9) mice reveal that treated mice behave like normal sighted mice (A, B). Average values for photopic visual acuity and contrast sensitivity of all GC1+/+ eyes (n = 4), untreated GC1KO eyes (n = 9) and AAV5-mGC1-treated eyes (n = 5) are shown (B) (* = *P*<0.0001). Cone-mediated ERG responses from each mouse (M1–M9) are shown for comparison (C).

Optomotor analysis revealed that eyes of GC1KO mice treated with either smCBA-mGC1 (Figure 5A: M5, M6, M7) or hGRK1-mGC1 (Figure 5: M8, M9) responded significantly better than untreated eyes under all photopic, cone-mediated conditions. Untreated GC1KO eyes perform poorly with a visual acuity of 0.163±0.040 cycles per degree (Figure 3B, 3C, blue bar, mean ± s.d., *n* = 9 eyes). Isogenic GC1+/+ control eyes (Figure 5: M1, M2) respond significantly better, showing an average acuity of 0.418±0.046 cycles per degree (black bar, *n* = 4 eyes). AAV-mGC1-treated eyes (Figure 5: M5–M9) have an average acuity of 0.392±0.077 cycles per degree (red bar, *n* = 5 eyes), a level essentially identical to control +/+ eyes and significantly better than untreated GC1KO eyes (P<0.0001). Photopic contrast sensitivities (Figure 5A, 5B) paralleled the photopic acuity results, with AAV-mGC1-treated eyes (contrast sensitivity of 11.90±7.37, *n* = 5 eyes) showing contrast thresholds identical to +/+ mice (11.94±3.03, *n* = 4 eyes). Again, GC1KO eyes treated at P14 with AAV-mGC1 performed significantly better than untreated eyes, exhibiting an average contrast sensitivity of 1.27±0.31 (*n* = 9, P<0.0001). In all photopic tests, untreated GC1KO eyes perform extremely poorly, essentially equivalent to having no cone-mediated function. Statistical comparisons of these measurements are shown in Table 1. Cone-mediated ERG traces of all GC1+/+ (M1, M2), GC1KO (M3, M4), smCBA-mGC1-treated (M5, M5, M7) and hGRK1-mGC1-treated (M8, M9) mice used in the optomotor analyses are shown in Figure 5C to relate visual function (optmotor behavior) to retinal function (ERG).

**Table 1:** Statistical comparison of the photopic visual functions of +/+, P14-treated and untreated GC1KO mouse eyes as measured by optomotor behavior.

**Photopic Acuity**				
		WT	Tx	no Tx
Number of Values		4	5	9
Mean		0.4183	0.3919	0.163
Standard Deviation		0.0456	0.07731	0.03954
		***P*** **-value**		
	WT vs Tx	0.5671	Not significant	
	WT vs no Tx	<0.0001	*	
	Tx vs no Tx	<0.0001	*	
**Photopic Contrast Sensitivity**				
		WT	Tx	no Tx
Number of values		4	5	9
Mean		11.94	11.16	1.27
Standard Deviation		3.03	7.37	0.31
		***P*** **-value**		
	WT vs Tx	0.4186	Not significant	
	WT vs no Tx	<0.0001	*	
	Tx vs no Tx	<0.0001	*	

Under photopic conditions, P14 treatment with either AAV5-smCBA-mGC1 or AAV5-hGRK1-mGC1 produces acuity and contrast sensitivity similar to normal GC1+/+ animals. Treated eyes have significantly better acuity and contrast sensitivity than untreated GC1KO eyes. Each mouse was tested for 4–6 trials per condition. Data from each animal was then averaged to obtain the means for each condition. The standard deviation listed is the standard deviation of the individual mouse means. P-values were calculated using an unpaired t-test. *P<0.0001.

Rod ERG amplitudes are partially preserved in the GC1KO mouse. Studies have shown that even very small ERG amplitudes can translate into robust visual behavior [13] and that ERG activity (particularly in mouse) does not always reliably predict behavioral performance [38]. In fact, some LCA2 patients who received AAV-RPE65 therapy were found to exhibit behavioral restoration despite a complete lack of ERG response [39]–[40]. Optomotor testing revealed that scotopic, rod-mediated visual acuities and contrast sensitivities of untreated GC1KO eyes are very similar to isogenic, +/+ controls (data not shown). For this reason, it was impossible to compare rod-mediated visual restoration in treated vs. untreated eyes using these behavior tests. It is likely therefore that any future studies of the effects of AAV-mediated mGC1 expression on rod function in this mouse model will have to be evaluated at the level of ERG.

### Cone photoreceptors are preserved in AAV-mGC1-treated GC1KO mice

**Figure 6:**
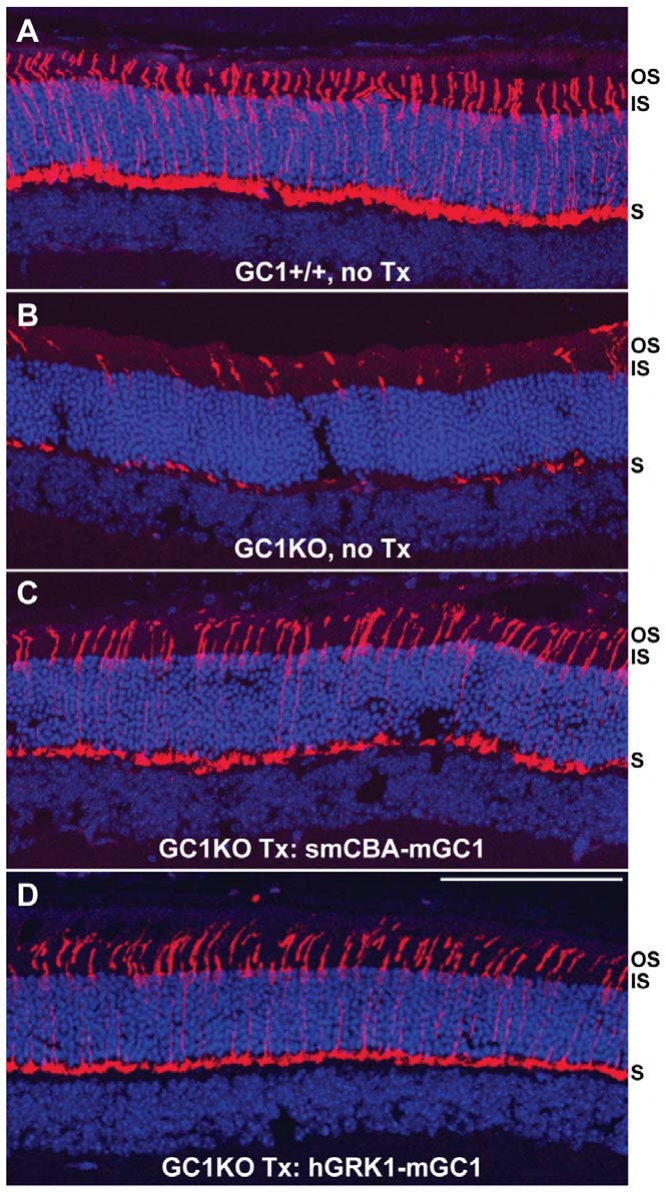
Cone arrestin expression in cone photoreceptors of +/+, GC1KO, AAV5-smCBA-mGC1-treated and AAV5-hGRK1-mGC1-treated mice. Untreated GC1KO retinas contain characteristic disorganized, detached cone outer segments (B), whereas cone outer segments were intact and cone arrestin distribution appeared normal in treated GC1KO (C,D) and +/+ (A) retinal sections. All retinas were taken from mice 3 months post treatment or age matched untreated controls. Scale bar in D = 100 µm. OS-outer segments, IS-inner segments, S-synaptic terminals.

**Figure 7:**
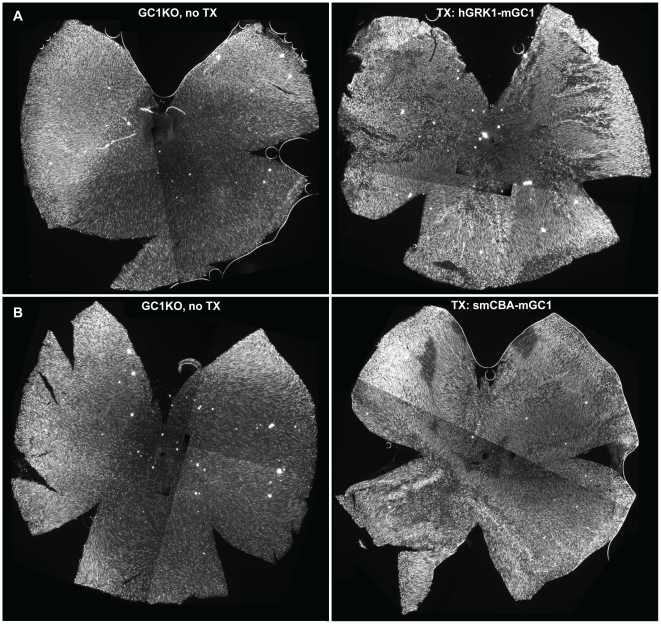
AAV-mediated GC1 expression preserves cone photoreceptors in GC1KO mice. Representative retinal whole mounts from AAV5-hGRK1-mGC1 treated (A, right), AAV5-smCBA-mGC1-treated (B-right) and contralateral, uninjected GC1KO eyes (A and B, left) stained for cone arrestin reveal that cone photoreceptors are preserved in GC1KO mice treated with AAV5-mGC1 for at least 3 months post treatment (the latest time point evaluated in this study). Cone cell densities were counted (see materials and methods) in central and inferior regions of treated and untreated GC1KO mice. Significant differences were found in both areas following treatment with either viral vector. All retinas were taken from mice 3 months post treatment.

Representative untreated GC1KO retinal sections immunostained with an antibody against cone arrestin revealed the characteristic disorganization and detachment of cone outer segments (Figure 6B). On the contrary, cone outer segments were intact and cone arrestin distribution appeared normal in treated GC1KO and +/+ retinal sections (Figure 6A,C,D, respectively). While not quantified in these retinal sections, these eyes also exhibit an apparent increase in cone cell densities relative to untreated controls. To quantify cone cell abundance, treated mice were sacrificed three months post-injection and retinal whole mounts from their treated and untreated eyes stained with an antibody against cone arrestin. Qualitatively, it appeared that cone photoreceptors were preserved as a result of treatment with both the smCBA-mGC1 and hGRK1-mGC1 vectors (Figure 7). Because cone cell loss progresses from central to peripheral retina (most notably inferior hemisphere) in the GC1KO mouse [18], we focused on comparing cone counts in these two areas. Cone cell quantification revealed a statistically significant difference in the cone densities of treated vs. untreated eyes. Cones were significantly more abundant in the central and inferior retinas of a representative hGRK1-mGC1-treated retina relative to untreated, contralateral control (*P*<0.001 and <0.001, respectively) (Figure 7A). Similar differences were seen in central and inferior areas of a representative smCBA-mGC1-treated retina and its contralateral control (*P* = 0.008 and <0.001, respectively) (Figure 7B). Raw cone cell counts from each respective whole mount are found in Table S1. P14- treatment of GC1KO mice with either therapeutic vector is therefore capable of preserving cone photoreceptor structure for at least three months (the longest time point evaluated in this study), a result we anticipated given the robust electrophysiological and behavioral restoration that also was observed.

## Discussion

This is the first demonstration of gene-based restoration of both visual function/vision-elicited behavior and cone preservation in a mammalian model of GC1 deficiency. Importantly, results were obtained using a well characterized, clinically relevant AAV vector. Our results build upon two previous attempts to rescue vision in animal models of GC1 deficiency, both of which reported incomplete success [13]–[14]. Our study differs in that the species-specific (murine) GC1 cDNA was incorporated into the therapeutic vector. Why bovine GC1 failed to restore retinal function or visual behavior to the GC1KO mouse in previous studies remains unclear.

Three ongoing clinical trials for Rpe65-LCA (LCA2) have demonstrated the ability to target a therapeutic transgene to the retinal pigment epithelium thereby restoring retinal function and visually-evoked behavior to patients [31],[39],[40],[45]. However, the need to efficiently target therapeutic vectors to photoreceptor cells is required for the treatment of many other retinal disorders. Restoration of visual behavior following cone-specific targeting of a therapeutic transgene has been reported in several animal models of cone dysfunction [33],[46]–[47]. Our findings add to this array of gene therapy tools because they are the first demonstration that visual behavior can be restored to an animal model following delivery of a gene targeted to both rod and cone photoreceptors, not just cones. This was achieved through the use of a serotype 5 AAV vector which has proven effective at targeting photoreceptors in a number of animal species including mouse, dog and monkey [33],[46]–[50]. Our methods differ from recent reports of restoration of retinal function in mouse models of AIPL1-LCA [36]–[37] and RPGRIP-LCA [51] which employed serotype 8 AAV as a means to confer therapy. While AAV5 has a slower onset of transgene expression than AAV8 [52], there is evidence that AAV8 has the increased potential for transfer across synapses which may limit its use for photoreceptor-targeted gene therapy in humans [53]. Our results suggest that the well-characterized AAV5 vector is sufficient for targeting therapy to photoreceptor cells.

Promoter choice is another vital component of any gene replacement strategy. For the purposes of targeting expression to photoreceptors, we chose to use the photoreceptor-specific hGRK1 promoter as well as the well-characterized, ubiquitous smCBA promoter. Both drove murine GC1 expression exclusively in photoreceptor cells. Similar to previous reports, hGRK1-mediated transgene expression was limited to photoreceptor cells [36]–[37]. More specifically, GC1 expression was found only in photoreceptorouter segments. smCBA-mediated GC1 expression was similar with an added rare presence of the transgene product in photoreceptor inner segments and cell bodies. However, like the hGRK1 vector, the majority of transgene expression was localized to photoreceptor outer segments. Neither promoter drove any off-target expression suggesting that any expression from the smCBA vector in RPE cells is negligible or very inefficient, perhaps due to the instability of mGC1 within the context of the incorrect retinal cell type. A recent study showed that, when used in conjunction with AAV, hGRK1 also efficiently drove AIPL1 expression exclusively in rod and cone photoreceptors of AIPL1 mutant mice [36]. Sun et al. (2010) suggest this promoter would be well-suited for human application. However, another report revealed that the specificity of hGRK1 promoter in canine retina is quite different [28]. hGRK1 drove transgene expression exclusively in rod photoreceptors of the canine retina, except when very high viral vector titers were used [28]. It was speculated that the discrepancy between murine and canine retina was due to a species-specific difference in expression of G-protein coupled receptor kinases in rods and cones. GRK1 is expressed in rods and cones of mice and rats, but is only found in rods of the canine retina [54]. Desensitization of cones in these species is modulated by GRK7, a cone-specific isoform [54]. In monkey and human retinas, GRK1 is localized to rods but is also co-expressed with GRK7 in cones [54]. We postulate that the lack of redundant GRK isoforms may be responsible for the high acitivity of hGRK1 in mouse cones [28]. However, the existence of these isoforms in human retina may limit the ability of hGRK1 to efficiently target both cell types. The activity of this promoter therefore deserves careful examination in the non-human primate retina. Our study also showed that the smCBA promoter drove no off-target transgene expression in the GC1KO retina. This is a truncated version of the chimeric CMV-chicken beta-actin promoter (CBA). CBA has been shown to be safe, effective and persistent when used in proof of concept experiments and in clinical trials of retinal disease [14],[31],[39]–[40],[45],[49], [55]–[56] and is perhaps better suited for translation into the clinic.

The GC1 null genotype produces a recessive cone dystrophy in the GC1KO mouse. LCA1 patients, in addition to having cone degeneration and extinction of photopic ERG responses also exihibit rod degeneration and extinction of scotopic ERGs. The phenotypic differences between mouse and man are thought to be attributed to a species-specific dependence on GC2, a cyclase proven to support rod function in the GC1KO mouse [22]. Indeed, a GC1/GC2 double knockout mouse phenotypically resembles human LCA1 [22]. Additional studies will be required to evaluate the effects of AAV-mediated GC1 expression in rod photoreceptors. While we observed some instances of increased rod-mediated ERG responses in treated eyes of the GC1KO mouse, this effect was not as consistent as that seen for cone responses upon treatment. It is possible that the treatment effect was less obvious in rods due to residual GC2-mediated function. For this reason, it could be informative to test our therapeutic construct(s) in the GC1/GC2 double knockout mouse. Regardless, it is likely that GC1 will need to be targeted to both cones and rods of LCA1 patients. We believe our clinically relevant smCBA-containing vector may be well suited for this task.

Finally, it is important to note that LCA1 patients maintain photoreceptor cell bodies for decades [6],[12] and therefore may have a relatively large age window for gene replacement therapy. In fact, maintenance of macular morphology in LCA1 patients is far better than that seen in LCA2 patients of similar age [12]. In addition, the number of patients affected by LCA1 is approximately double that affected by LCA2 [8], [9]. The results of three ongoing clinical trials for LCA2 have revealed safety and efficacy [39]–[40], [45] with some patients exhibiting visual gains sufficient to generate a new locus of fixation or pseudo-fovea [55]. The fact that some LCA2 patients treated with AAV-RPE65 exhibited profound improvements despite the relatively poor integrity of their retinas [55] argues strongly for the development of an AAV-based therapy for treating LCA1. The improved structural integrity of their retinas relative to other LCA patients suggests that they may have even more to gain from gene replacement therapy.

## Supporting Information

Table S1 Cone cell counts in retinal whole mounts from hGRK1-mGC1-treated, smCBA-mGC1-treated and untreated GC1KO mouse eyes. Five samplings were taken from identical areas of each central/inferior retina; values were averaged and standard deviations calculated. P values were calculated between respective groups. Standard t-tests were used to compare densities in each eye. Significant difference was defined as P<0.05.
(9.29 MB TIF)
