# Supplementary figures and images for: Functional and Behavioral Restoration of Vision by Gene Therapy in the Guanylate Cyclase-1 (GC1) Knockout Mouse
Source: PLoS One. 2010 Jun 25;5(6):e11306. doi: 10.1371/journal.pone.0011306 (PMC2892468; doi:10.1371/journal.pone.0011306)

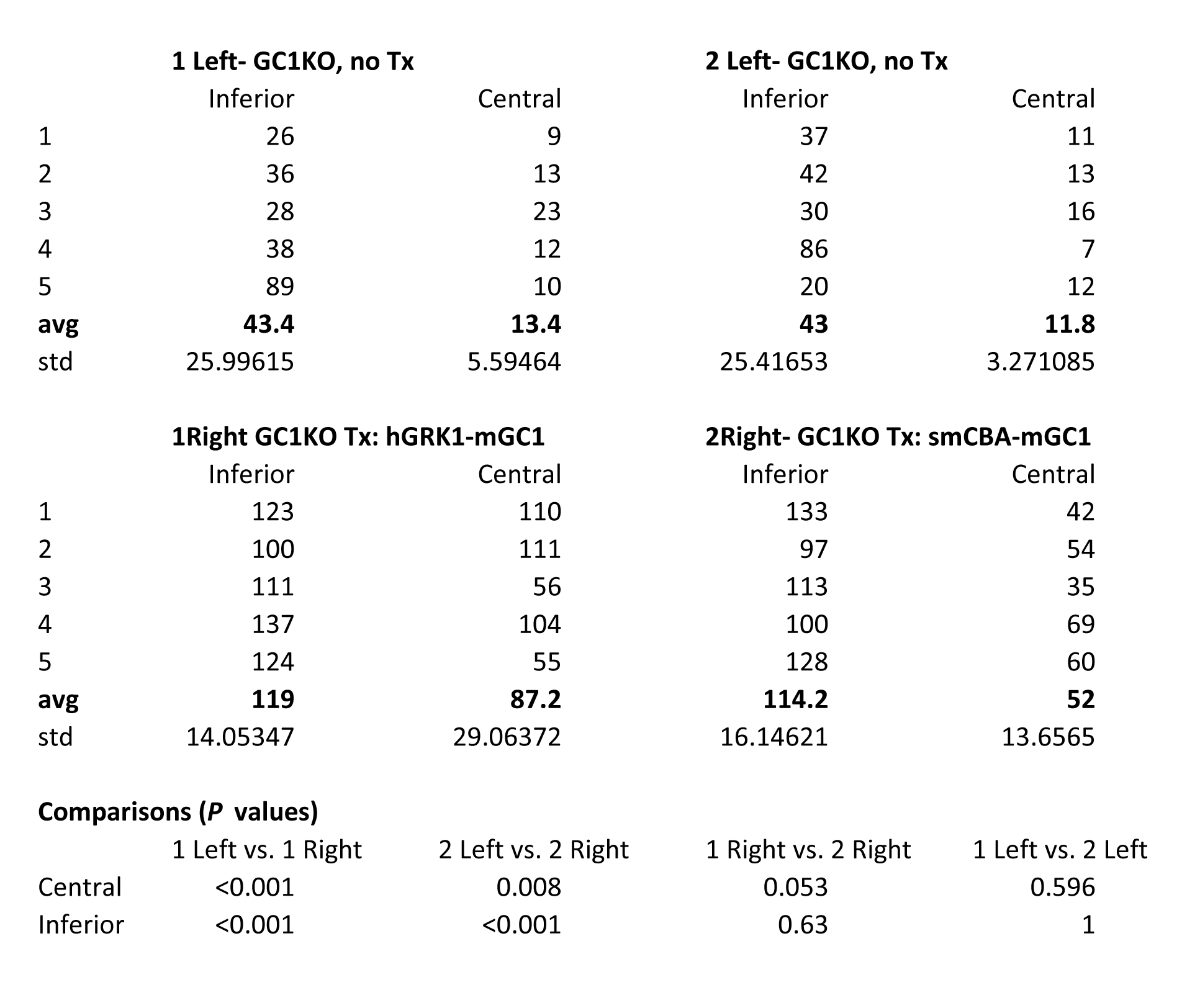

Supplement: Table S1 — Cone cell counts in retinal whole mounts from hGRK1-mGC1-treated, smCBA-mGC1-treated and untreated GC1KO mouse eyes. Five samplings were taken from identical areas of each central/inferior retina; values were averaged and standard deviations calculated. P values were calculated between respective groups. Standard t-tests were used to compare densities in each eye. Significant difference was defined as P<0.05. (9.29 MB TIF) [file pone.0011306.s001.tif]
